# Risk-stratified surveillance protocol improves cost-effectiveness after radical nephroureterectomy in patients with upper tract urothelial carcinoma

**DOI:** 10.18632/oncotarget.25198

**Published:** 2018-05-01

**Authors:** Masaki Momota, Shingo Hatakeyama, Hayato Yamamoto, Hiromichi Iwamura, Yuki Tobisawa, Tohru Yoneyama, Takahiro Yoneyama, Yasuhiro Hashimoto, Takuya Koie, Ikuya Iwabuchi, Masaru Ogasawara, Toshiaki Kawaguchi, Chikara Ohyama

**Affiliations:** ^1^ Department of Urology, Hirosaki University Graduate School of Medicine, Hirosaki, Japan; ^2^ Department of Urology, Tohoku Medical and Pharmaceutical University, Sendai, Japan; ^3^ Department of Advanced Transplant and Regenerative Medicine, Hirosaki University Graduate School of Medicine, Hirosaki, Japan; ^4^ Department of Urology, Aomori Prefectural Central Hospital, Aomori, Japan

**Keywords:** upper tract urothelial carcinoma, radical nephroureterectomy, recurrence, surveillance, cost-effectiveness

## Abstract

**Objectives:**

To develop a surveillance protocol with improved cost-effectiveness after radical nephroureterectomy (RNU), as the cost-effectiveness of oncological surveillance after RNU remains unclear.

**Results:**

Of 426 patients, 109 (26%) and 113 (27%) experienced visceral and intravesical recurrences, respectively. The pathology-based protocol found significant differences in recurrence-free survival in the visceral recurrence but not in the intravesical recurrence. The medical costs per visceral recurrence detected were high, especially in normal-risk (≤ pT2N0, LVI-, SM-) patients. We developed a risk score associated with visceral recurrence using Cox regression analysis. The risk score-based protocol was significantly more cost-effective than the pathology-based protocol. Estimated cost differences reached $747,929 per recurrence detected, a suggested 55% reduction.

**Materials and Methods:**

We retrospectively evaluated 426 patients with RNU for upper tract urothelial carcinoma (UTUC) without distant metastasis at 4 hospitals. Patients with routine oncological follow-up were stratified into normal-, high- and very high-risk groups according to a pathology-based protocol utilizing pathological stage, lymphovascular invasion (LVI) and surgical margin (SM). Cost-effectiveness of the pathology-based protocol was evaluated, and a risk score-based protocol was developed to optimize cost-effectiveness. Risk scores were calculated by summing up risk factors independently associated with recurrence-free survival. Patients were stratified by low-, intermediate- and high-risk score. Estimated cost per recurrence detected by pathology-based and risk score-based protocols was compared.

**Conclusions:**

A risk score-stratified surveillance protocol has the potential to reduce over investigation during follow-up, making surveillance more cost-effective.

## INTRODUCTION

Upper tract urothelial carcinoma (UTUC) is relatively rare, and radical nephroureterectomy (RNU) with bladder cuff excision remains the standard treatment modality for non-metastatic UTUC [1]. The prognosis of patients with locally advanced UTUC has not improved over the past 2 decades [2–6] despite established predictors of prognosis in patients with UTUC including older age, tumor stage, presence of hydronephrosis, tumor location, lymphovascular invasion (LVI), surgical margin (SM) and lymph node involvement [3, 7–10]. In addition, the effective follow-up frequency and evaluation methods remain unclear, although various surveillance regimens have been proposed for muscle-invasive bladder cancer [11–15]. Furthermore, there is a lack of evidence regarding the cost-effectiveness of routine oncological follow-up to detect recurrence after RNU. Therefore, we aimed to develop the optimal surveillance protocol to improve cost-effectiveness after RNU. First, we estimated the per person cost of detecting post-RNU recurrence using our pathology-based protocol. Further, we developed a novel risk score-based protocol using Cox proportional hazards regression and compared the cost-effectiveness of the pathology- and risk score-based protocols.

## RESULTS

### Background of patients

Of 426 patients with RNU, 109 (26%) and 113 (27%) experienced visceral and intravesical recurrences, respectively. Of these, 209 (49%), 182 (43%), and 35 (8.2%) were included in the pathology-based normal-risk, high-risk, and very high-risk group, respectively. The clinicopathological characteristics are shown in Table 1. Pathological outcomes (≥ pT3, LVI+, SM+) were significantly worse in high- and very high-risk patients compared to normal risk-patients. There were no significant differences in clinical parameters except for ≥ cT3, cN+, and presence of hydronephrosis between high-/very high-risk and normal risk-patients.

**Table 1:** Background of patients

*n*	426
Age (years)	70 ± 8.9
Sex (Male), *n* =	290 (68%)
ECOG-PS > 1, *n* =	10 (2.3%)
Hypertension, *n* =	185 (43%)
Diabetes Mellitus (DM), *n* =	70 (16%)
Cardiovascular disease (CVD), *n* =	75 (18%)
Smoking, *n* =	193 (45%)
eGFR before surgery (ml/min/1.73 m^2^)	58 ± 18
Hydronephrosis, *n* =	266 (62%)
Neoadjuvant chemotherapy (NAC), *n* =	102 (24%)
Clinical stage	
≥ cT3, *n* =	229 (54%)
cN+, *n* =	34 (8.0%)
Tumor location, *n* =	
Renal pelvis	166 (39%)
Ureter	235 (55%)
Multiple	25 (5.9%)
Laparoscopic surgery, *n* =	75 (18%)
Postoperative complications (G3 or higher), *n* =	14 (3.3%)
Pathological outcome, *n* =	
≥ pT3	182 (43%)
pN+	30 (7.0%)
High grade	397 (93%)
Surgical margin (SM) positive	14 (3.3%)
Lymphovascular invasion (LVI) positive	127 (30%)
Adjuvant Chemotherapy, *n* =	44 (10%)
Median follow-up (Months)	40
Disease recurrence, *n* =	
Intravesical	113 (27%)
Visceral	109 (26%)
Deceased, *n* =	
Cancer-specific	80 (19%)
Any cause	103 (24%)

### Oncological and economic outcomes of pathology-based surveillance protocols

**Figure 1:**
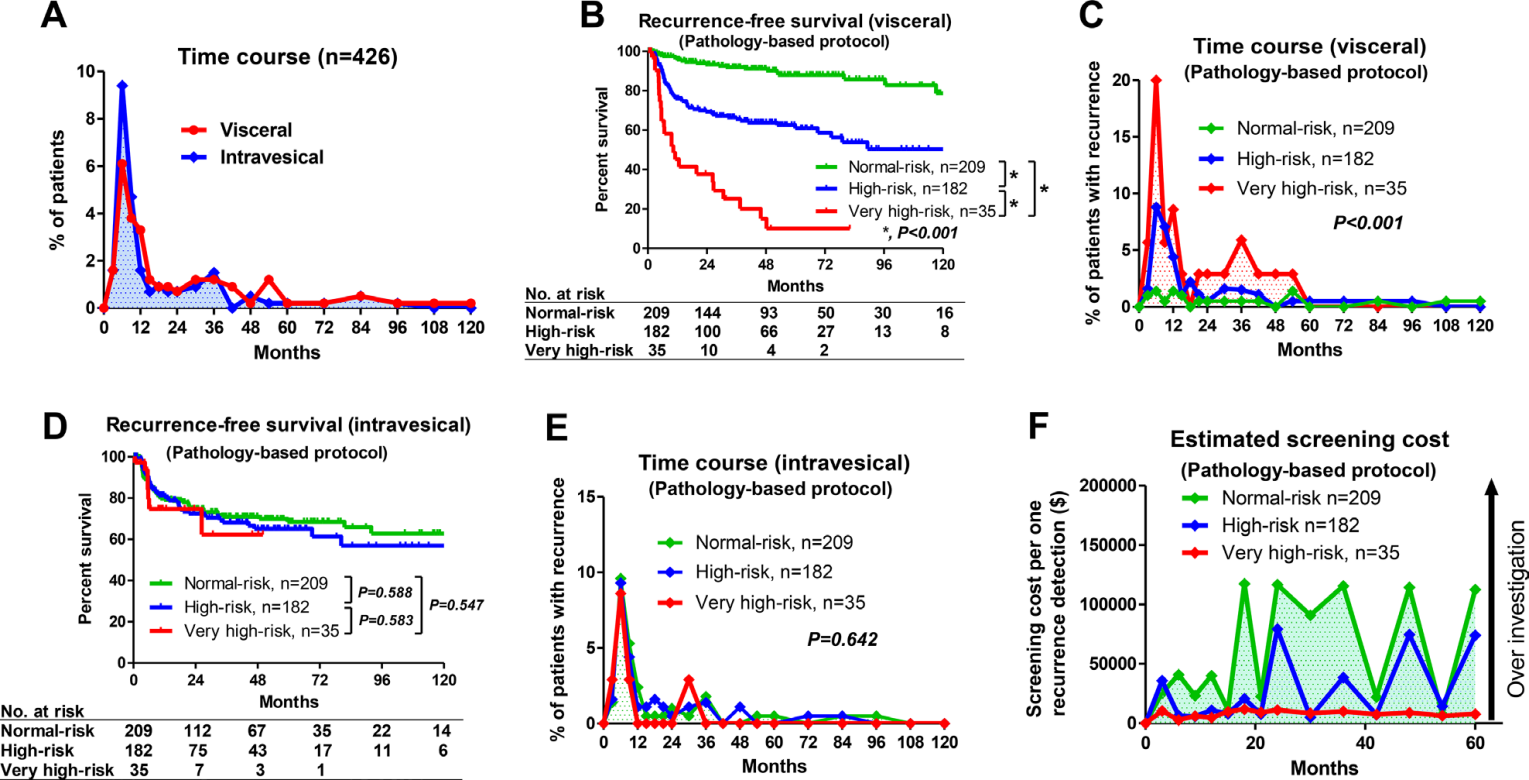
Oncological and economic outcomes of pathology-based surveillance protocols (**A**) Time-course analysis of visceral and intravesical recurrence patterns. Number of patients with visceral and intravesical recurrences within 3 years after radical nephroureterectomy was 88/109 (81%) and 96/113 (85%), respectively. (**B**) Visceral recurrence-free survival in normal-risk (≤ pT2N0, lymphovascular invasion- [LVI-], surgical margin- [SM-], high-risk [N0 with pT3 or LVI+]), and very high-risk (pT4, SM+, or N+) patients in the pathology-based protocol. There were significant differences in prognosis among the groups (*P* < 0.001). (**C**) Time-course analyses of visceral recurrence patterns of normal-, high- and very high-risk patients in the pathology-based protocol. There were significant differences in visceral recurrence patterns among the groups (*P* < 0.001). (**D**) Intravesical recurrence-free survival in normal-, high- and very high-risk patients in the pathology-based protocol. There were no significant differences in intravesical recurrence among the groups (*P* = 0.722). (**E**) Time-course analyses of intravesical recurrence patterns of normal-, high- and very high-risk patients in the pathology-based protocol. There were no significant differences in intravesical recurrence patterns among the groups (*P* = 0.642). (**F**) Estimated cost per detected recurrence in normal-, high- and very high-risk patients in the pathology-based protocol. Estimated costs for patients with normal risk were high (> $50,000) after 18 months.

Time course of intravesical and visceral recurrences in all patients was shown in Figure 1A. Most patients experienced visceral (*n* = 79/109; 72%) and intravesical (*n* = 87/113; 80%) recurrences within 24 months. Visceral recurrence-free survival in the 3 groups was also significantly different (*P* < 0.001; Figure 1B). The number of patients with visceral recurrence within 24 months was significantly different among patients with normal-risk (*n* = 13/209; 6.2%), high-risk (*n* = 49/182; 27%), and very high-risk (*n* = 17/35; 49%) (*P* < 0.001; Figure 1C). On the other hand, intravesical recurrence-free survival in the 3 groups was not significantly different (Figure 1D). The number of patients with intravesical recurrence within 24 months was not significantly different among patients with normal risk (*n* = 44/209; 21%), high risk (*n* = 38/182; 27%), and very high risk (*n* = 5/35; 14%) (*P* = 0.642; Figure 1E). The estimated cost per detected recurrence was acceptable within 18 months in the normal-risk and high-risk group (< $50,000) but it increased thereafter (Figure 1F). The cost per detected recurrence was very low in the very high-risk group for 5 years (< $10,000).

### Oncological and economic outcomes of risk score-based surveillance protocols

**Figure 2:**
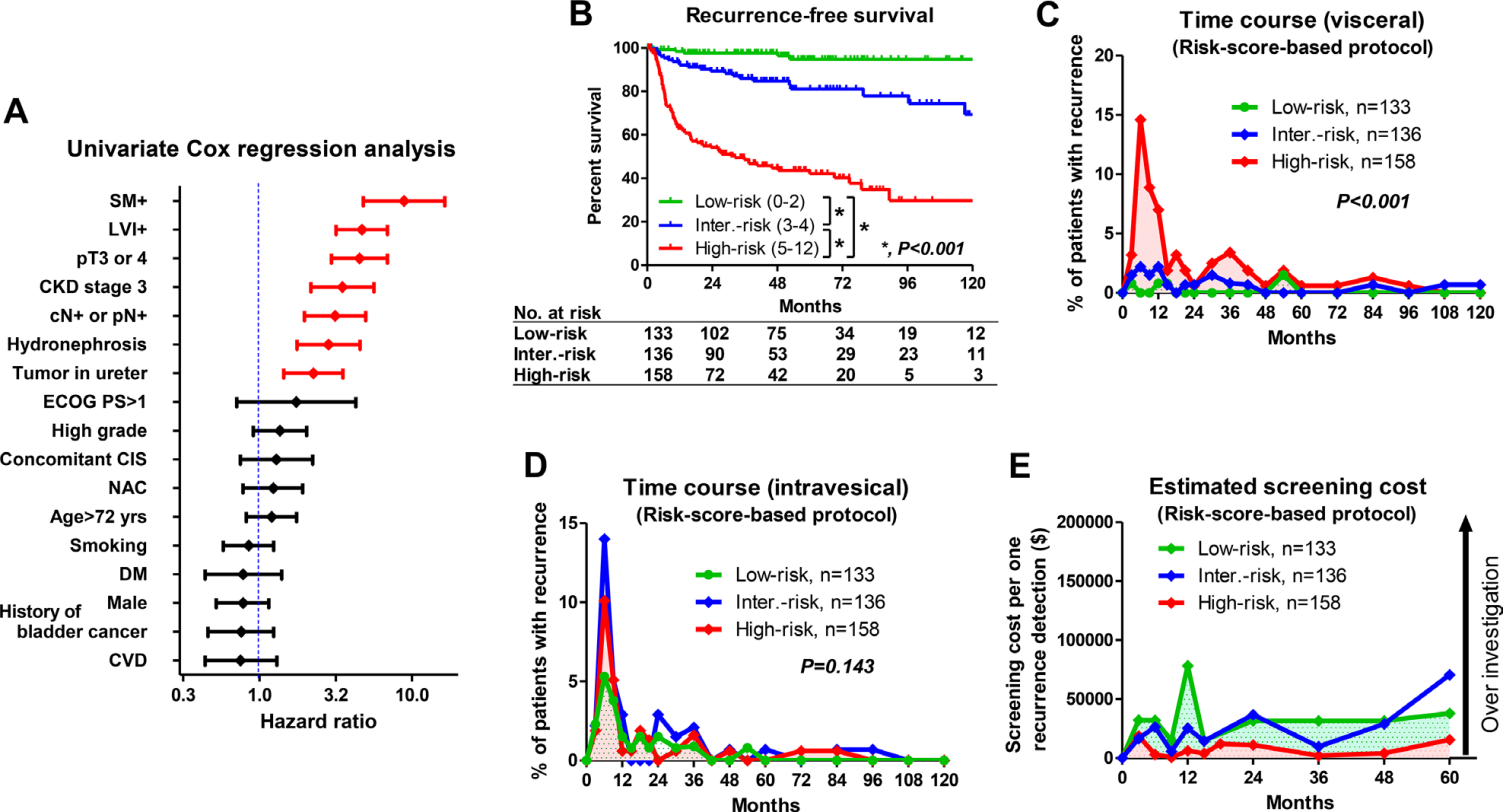
Oncological and economic outcomes of risk-score-based surveillance protocols (**A**) Seven independent risk factors for visceral recurrence were selected using univariate analysis after radical nephroureterectomy (RNU). Risk scores were calculated using multivariate Cox regression analysis using tumor in ureter (score 1), presence of hydronephrosis (score 1), lymph node involvement (score 2), preoperative CKD (score 2), pT3-4 (score 2), LVI+ (score 2) and SM+ (score 2). (**B**) Visceral recurrence-free survival was shown for each risk score. Patients were stratified into 3 groups by low- (0–2), intermediate- (3–4) and high-risk scores (5–12), indicating the probability of relapse. There were significant differences in prognosis among the groups (*P* < 0.001). (**C**) Time-course analyses of recurrence in low-, intermediate- and high-risk scores showed that the number of patients with visceral recurrence in the high-risk group was significantly higher compared to those in the low- and intermediate-risk groups. (**D**) Intravesical recurrence-free survival was shown for each risk score. There were no significant differences in intravesical recurrence among the groups (*P* = 0.143). (**E**) The estimated cost per detected recurrence was acceptable and estimated to be within $50,000 for 5 years in all risk score groups except for the low-risk score patients at 12 months and the intermediate-risk score patients at 60 months. SM: surgical margin, LVI: lymphovascular invasion, CKD: chronic kidney disease, CVD: cardiovascular disease, HTN: hypertension, NAC: neoadjuvant chemotherapy, DM: diabetes mellitus. CIS: carcinoma *in situ*.

Uni- and multivariate Cox regression analysis found 7 factors that were independently associated with recurrence-free survival, including SM+ (score 2), LVI+ (score 2), ≥ pT3 or 4 (score 2), preoperative stage 3 CKD (score 2), cN+ or pN+ (score 2), hydronephrosis (score 1), and tumor location (score 1) (Table 2, Figure 2A). When hazard ratio was greater than 3, the score was marked as 2. The risk scores were calculated by adding each independent risk factor for recurrence-free survival (risk score ranged 0–12). Patients were divided by their score into low-risk (0–2), intermediate-risk (3–4), and high-risk (5–12) groups (Table 2). Recurrence-free survival was significantly shorter in patients with high-risk than with low-risk or intermediate-risk (*P* < 0.001) scores (Figure 2B). The number of patients who experienced visceral recurrence within 24 months was significantly higher in those with high-risk (*n* = 65/158; 41%) versus low-risk (*n* = 3/133; 2.3%) or intermediate-risk scores (*n* = 13/136, 9.6%) (*P* < 0.001; Figure 2C). However, the number of patients with intravesical recurrence within 24 months was not significantly different among those with low-risk (*n* = 23/133; 17%), intermediate-risk (*n* = 37/136, 27%), and high-risk scores (*n* = 34/158; 22%) (*P* = 0.143) (Figure 2D). Based on this risk stratification, we developed a risk score-based protocol for oncological follow-up (Table 3) and evaluated per person cost of visceral recurrence detection. The estimated cost per detected recurrence was acceptable and estimated within $50,000 for 5 years in all risk score groups except for the low-risk score patients at 12 months and the intermediate-risk score patients at 60 months (Figure 2E).

**Table 2:** Multivariate analysis for risk score calculation

Factor	*P* value	HR	95% CI	Risk score
Tumor in ureter	< 0.001	2.25	1.44–3.52	1
Hydronephrosis	< 0.001	2.83	1.76–4.56	1
Lymph node involvement (cN+ or pN+)	< 0.001	3.13	1.97–4.97	2
Preoperative CKD	< 0.001	3.49	2.17–5.62	2
pT3-4	< 0.001	4.52	2.96–6.89	2
LVI+	< 0.001	4.68	3.18–6.89	2
SM+	< 0.001	8.85	4.78–16.4	2

CKD: chronic kidney disease, LVI: lymphovascular invasion, SM: surgical margin.

**Table 3:** Risk-score-based protocol

Risk-score-based	Months after RNU													
Type of investigation	3	6	9	12	15	18	21	24	30	36	42	48	54	60
Basic exam (blood and serum test, ultrasound, and chest X-ray)	●	●		●		●		●		●		●		●
Urine analysis, cytology and cystoscope	●	●	●	●	●	●		●		●		●		●
CT scan of chest/abdomen/pelvis														
Low-risk (0–2)				●										●
Intermediate-risk (3–4)		●		●				●		●		●		●
High-risk (5–12)	●	●	●	●	●	●		●		●		●		●

RNU: radical nephroureterectomy.

### Time course differences in patients with visceral recurrence (*n* = 109)

**Figure 3:**
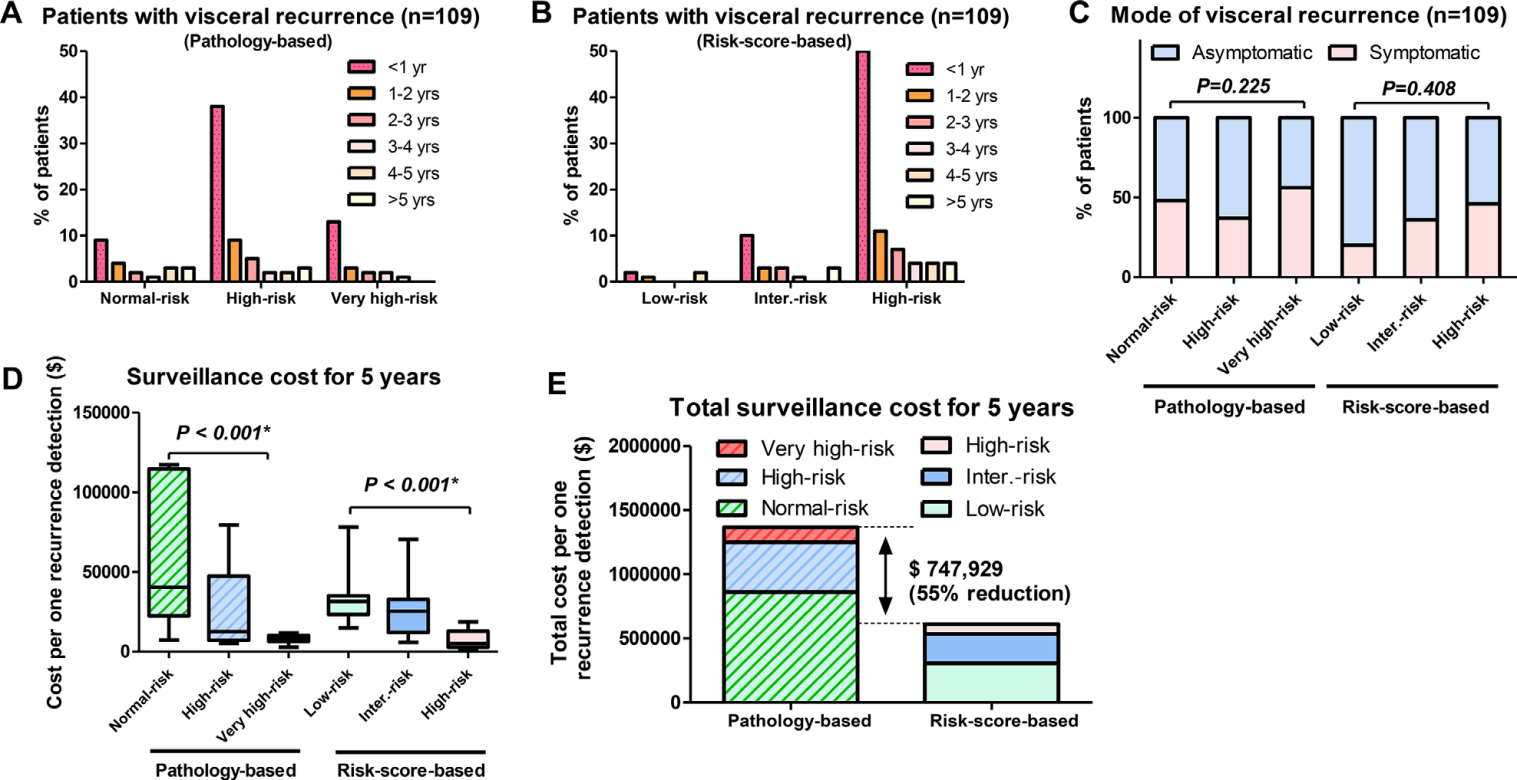
Time course and cost differences between the pathology-based and risk-score-based surveillance protocols (**A**) The pathology-based protocol showed higher incidence of visceral recurrence within 1 year in the high-risk (38%) than in the normal-risk (9%) and very high-risk (13%) groups. (**B**) The risk score-based protocol showed higher incidence of visceral recurrence within 1 year in patients with high-risk (50%) than in those with low-risk (2%) and intermediate-risk (10%) scores. (**C**) The number of patients with symptomatic recurrence in the normal-, high-, and very high-risk groups was 10 of 21 (48%), 23 of 63 (37%), and 14 of 25 (56%) (*P* = 0.225). The number of patients with symptomatic recurrence in the low-, intermediate-, and high-risk groups was 1 of 5 (20%), 8 of 22 (36%), and 38 of 82 (46%), respectively (*P* = 0.408). (**D**) In the pathology-based protocol, median surveillance cost to detect 1 recurrence in 5 years was significantly different in the 3 groups (*P* < 0.001, Kruskal–Wallis test). In the risk score-based protocol, median surveillance cost to detect 1 recurrence in 5 years was significantly different in the 3 groups (*P* < 0.001, Kruskal–Wallis test). (**E**) The total estimated 5-year surveillance cost was 2.2-fold higher with the pathology-based protocol ($1,365,245) than with the risk score-based protocol ($617,315). The estimated cost difference was $747,929 for 5 years, a suggested 55% cost reduction.

The risk score-based protocol effectively stratified patients with visceral recurrence compared to the pathology-based protocol. The pathology-based protocol showed higher incidence of visceral recurrence within 1 year in the high-risk (38%) than in the normal-risk (9%) and very high-risk (13%) groups (Figure 3A). However, the risk score-based protocol showed higher incidence of visceral recurrence within 1 year in patients with high-risk (50%) than in those with low-risk (2%) and intermediate-risk (10%) scores (Figure 3B). In total, 47 of 109 patients had symptomatic visceral recurrence. The number of patients with symptomatic recurrence in the normal-, high-, and very high-risk groups was 10 of 21 (48%), 23 of 63 (37%), and 14 of 25 (56%) (*P* = 0.225; Figure 3C). The number of patients with symptomatic recurrence in the low-, intermediate-, and high-risk groups was 1 of 5 (20%), 8 of 22 (36%), and 38 of 82 (46%), respectively (*P* = 0.408; Figure 3C).

### Economic outcome differences between the pathology- and risk score-based protocols

In the pathology-based protocol, median surveillance cost to detect 1 recurrence in 5 years was significantly different in the 3 groups (*P* < 0.001, Kruskal–Wallis test; Figure 3D). Median estimated surveillance costs per detected visceral recurrence were $40,501, $18,775, and $8,622 in the normal-, high-, and very high-risk groups. In the risk score-based protocol, median surveillance cost to detect 1 recurrence in 5 years was significantly different in the 3 groups (*P* < 0.001, Kruskal–Wallis test, Figure 3D). The median estimated surveillance costs per detected visceral recurrence were $31,551, $25,486, and $5,140 in the low-, intermediate-, and high-risk score groups, respectively. The risk score-based protocol led to a dramatic cost reduction compared to the pathology-based protocol. The total estimated 5-year surveillance cost was 2.2-fold higher with the pathology-based protocol ($1,365,245) than with the risk score-based protocol ($617,315). The estimated cost difference was $747,929 for 5 years (Figure 3E), a suggested 55% cost reduction.

## DISCUSSION

There is no strong evidence for an optimal follow-up protocol in UTUC patients after RNU [5, 6, 8], and no studies have been conducted to investigate the cost-effectiveness of surveillance regimens after RNU. Although pathological stage is known to be a strong predictor of relapse, heterogeneity in UTUC patients prevents effective surveillance with a universal protocol for all patients. Our results showed that a pathology-based surveillance protocol successfully stratified the risk of visceral recurrence but potentially increased unnecessary testing in normal-risk (≤ pT2N0, LVI-, SM-) patients. Because pathological outcome and other clinical risk factors have a significant impact on tumor recurrence after RNU, we developed a risk score-based surveillance protocol including clinical risk factors and stratified the study patients into low-risk (0–2), intermediate-risk (3–4) and high-risk (5–12) score groups. As shown in Figure 2A, pathological outcomes and preoperative variables, including presence of stage 3 chronic kidney disease (CKD), hydronephrosis, and ureter/multiple tumor, had a significant impact on recurrence after RNU. The reason for the strong association between CKD and poor recurrence-free survival is not clear. Previous studies have suggested that kidney and urinary tract cancers are high risk for renal dysfunction due to tumor location (obstruction, and/or reduction of nephron mass) [16–18]. Our previous studies suggested that patients with stage 3 CKD had significantly higher risk for recurrence and cancer death compared to those without CKD [19–21]. In addition, UTUC has great potential for renal dysfunction during disease progression. Presence of hydronephrosis was an independent factor for recurrence in this study. Close relationship exists between presence of locally advanced UTUC, hydronephrosis, and renal impairment. Future studies to assess the underlying mechanisms of carcinogenesis and renal impairment are warranted.

Conversely, we could not address the effectiveness of a risk score-based protocol in intravesical recurrence. In univariate Cox regression analysis, previous/concomitant bladder cancer (*P* = 0.005) and stage 3 CKD (*P* = 0.008) were independent factors for intravesical recurrence (Supplementary Figure 1A). Although presence of hydronephrosis was not significant (*P* = 0.062), we included it as a risk score calculation due to its marginal influence on intravesical recurrence. Intravesical recurrence-free survival was significantly poor in patients with risk scores of 2–3 (high risk) compared to those with risk scores of 0–1 (low risk) (Supplementary Figure 1B; *P* = 0.001). However, the time course of intravesical recurrence between the low- and high-risk scores was not significantly different (Figure 2D). These results suggested that risk stratification for intravesical recurrence might not be useful. In addition, 91% (103/119) of patients experienced intravesical recurrence within 36 months. Therefore, routine surveillance such as cystoscope and urine cytology every 3–6 months within 36 months might be optimal for detecting intravesical recurrence.

Our results suggested a potential benefit of this risk score-based protocol to reduce over investigation after RNU. However, the cost was substantial as the low relapse rate in patients with low-risk scores increased the surveillance cost, highlighting the limitation of predicting relapse risk based exclusively on clinicopathological information. In addition, prediction of symptomatic recurrence is challenging. Several reports have suggested that presence of symptom at tumor recurrence is an independent predictor for poor prognosis, and detection of recurrence at the asymptomatic stage may improve prognosis [13, 22, 23]. However, our results showed that both pathology- and risk score-based stratification failed to separate the mode of recurrence. These results suggested that molecular biomarkers such as genomic subtypes in patients with symptomatic recurrence need to be investigated further to improve clinical management [24, 25].

This study was limited by its retrospective design. First, we could not control the influence of all confounding factors, selection bias, and medical costs for oncological surveillance. Actual surveillance protocol might be not completely same in four different hospitals due to a retrospective manner. Selection biases exist for lymph node dissection and adjuvant chemotherapy administration in the present study. Recently, a phase III randomized trial of perioperative chemotherapy versus surveillance in UTUC (POUT trial) suggested benefit of adjuvant chemotherapy for disease free survival (DFS) for patients with histologically confirmed pT2-T4 N0-3 M0 UTUC [26]. Therefore, adjuvant chemotherapy (four cycles of gemcitabine-cisplatin or gemcitabine-carboplatin if estimated glomerular filtration rate [eGFR] 30−49 ml/min) will be standard therapy shortly. Second, the risk scoring in the present study might not be optimal for risk stratification. The hazard ratio difference between lymph node involvement and surgical margin was more than two folds. However, when we stratified based on actual hazard ratio into scoring system, we found no big difference in risk stratification and outcomes. Therefore, we used simple scoring system in the present study. Third, pathological risk stratification of pT2 patients into the normal-risk group might not be suitable. It is reported that pT2 disease has a high risk of recurrence in Japanese population [27]. However, patients with pT3 disease has much higher risk for disease recurrence than those with pT2 disease. In the present study, 2-year recurrence free survivals in pT1, pT2, pT3 and pT4 were 96%, 85%, 65%, and 18%, respectively. The difference in 2-year recurrence-free survival between pT1 and pT2 disease was smaller (11%; *P* = 0.009) compared with between pT2 and pT3 (20%; *P* < 0.001). In addition, pT2 patients who experienced disease recurrence had other significant risk factors such as LVI+/pN+, stage 3 CKD, and preoperative hydronephrosis. There were significant differences in the number of pT2 patients between with and without recurrence in LVI+/pN+ status (37% vs 16%; *P* = 0.034), stage 3 CKD (95% vs. 52%; *P* < 0.001), preoperative hydronephrosis (95% vs. 58%; *P* = 0.002). Therefore, pathological factor of pT2 alone was not strong predictor for disease recurrence. Based on these findings, we classified pT2 as a pathological low-risk group in the present study and developed risk-stratified protocol to overcome the limitation of pathological outcome. Fourth, the implication of this study may not be suitable for non-Japanese population. In addition, the risk score-based surveillance model might increase the number of patients not detected by surveillance. The estimated number of patients who potentially experienced the delay of detection and mean periods of delay were three patients (2.3%) and 5.2 months in the risk-score-based low-risk group, nine patients (6.6%) and 2.7 months in the risk-score-based intermediate-risk group, and 15 patients (9.5%) and 1.1 months in the risk-score-based high-risk group, respectively. In total, the estimated number of patients with detection delay and mean periods of delay were 27 patients (6.3%) and 1.7 months, respectively. Therefore, validation of both pathology-based and risk-score-based risk classification is needed. Despite these limitations, our study may give the impression that risk stratification in each nation is necessary to improve cost-effectiveness in each medical system. A prospective study on the cost-effectiveness of follow up using a universal, standard, and easily applicable surveillance model is required to validate these results.

In conclusion, a risk score-based surveillance protocol has the potential to reduce over investigation during follow-up, making surveillance more cost-effective. Further study is needed to determine the impact of risk stratification on the cost-effectiveness of oncological follow-up after RNU.

## MATERIALS AND METHODS

### Patient selection and variables

Between May 1995 and February 2017, 426 consecutive adults underwent RNU at Hirosaki University Hospital, Aomori Rosai Hospital, Mutsu General Hospital, and Aomori Prefectural Central Hospital. Inclusion criteria were UTUC patients without distant metastasis who underwent RNU. Exclusion criteria were lack of sufficient variables recorded in the chart including clinicopathological and oncological outcomes. All patients were treated with RNU with or without perioperative systemic chemotherapy. Based on pathological outcome after RNU, all patients were stratified for oncological follow-up to normal-risk (≤ pT2N0), high-risk (N0 with pT3 or LVI+) and very high-risk (pT4, SM+, or lymph node involvement [cN+ or pN+]) groups. The variables analyzed were age, sex, Eastern Cooperative Oncology Group Performance Status (ECOG PS), smoking, clinical stage, preoperative CKD (eGFR < 60 mL/min/1.73m^2^) [28], hydronephrosis, history of hypertension (HTN), cardiovascular disease (CVD), and diabetes mellitus (DM). Tumor stage and grade were defined by the 2009 TNM classification [29]. Postoperative complications were evaluated by the Clavien–Dindo classification [30]. This retrospective study was approved by the Ethics Committee of the Hirosaki University School of Medicine (authorization numbers 2017–089) including all hospitals.

### Surgical procedure

Open or laparoscopic nephroureterectomy, which includes the removal of kidney, ureter, and ipsilateral bladder cuff, was performed [1, 31]. The distal ureter was managed by the extravesical approach. Because lymph node dissection for UTUC is still controversial and appropriate dissection area has never been established yet, we underwent regional lymph node dissection or sampling was performed depending on tumor stage. When an obvious nodule existed, regional lymph node dissection was performed. When patients had ≥ cT2 disease without lymph node involvement, lymph node sampling was performed in selected patients. We did not use early (within 48 h) intravesical chemotherapy after RNU.

### Systemic chemotherapy

We have performed 2–4 courses of platinum-based neoadjuvant chemotherapy (NAC) for the treatment of locally advanced UTUC (cT3-4 and/or cN+) in selected patients. Regimens were selected based on guidelines regarding eligibility for the proper use of cisplatin [32] and the patient’s overall status. Adjuvant chemotherapy was administered in selected patients (high- or very high-risk patients with suitable general health status). Salvage chemotherapy after recurrence consisted of a platinum-based combination regimen. Regimens were selected based on residual renal function and overall status.

### Surveillance protocol

Oncological follow-up after RNU was performed following the several guidelines and previously published pathology protocols [14, 33]. The pathology-based protocol is shown in Table 4. Evaluations by bone scans or brain imaging were performed when clinically indicated. Visceral organ recurrence included metastasis to the liver, lungs, adrenal glands, and other intra-abdominal organs.

**Table 4:** Pathology-based protocol

Pathology-based	Months after RNU													
Type of investigation	3	6	9	12	15	18	21	24	30	36	42	48	54	60
Basic exam (blood and serum test, ultrasound, and/or chest X-ray)	●	●		●		●		●		●		●		●
Urine analysis, cytology and cystoscope	●	●	●	●	●	●	●	●	●	●	●	●	●	●
CT scan of chest/abdomen/pelvis														
Normal-risk (≤ pT2N0, LVI-, SM-)		●		●		●		●		●		●		●
High-risk (N0 with pT3, or LVI+)	●	●	●	●		●		●		●		●		●
Very high-risk (pT4, SM+, or N+)	●	●	●	●	●	●	●	●	●	●	●	●	●	●

RNU: radical nephroureterectomy, LVI: lymphovascular invasion, SM: surgical margin.

### Outcome measurements

Recurrence-free survival, time to recurrence, and estimated cost per recurrence detected by the pathology-based protocol were recorded for the normal-, high-, and very high-risk groups. To estimate cost-benefit, we calculated the medical cost of follow-up to detect 1 recurrence ([surveillance cost in a follow-up period] / [number of patients with recurrence]) using an exchange rate of 100 yen to the U.S. dollar. Estimated medical costs were $350 for computed tomography (CT), $70 for blood testing, $53 for ultrasonography, and $25 for urine cytology. The cost of prescribing medications, and doctor fees were not included in the analysis. A total examination fee was calculated as a total patient’s fee (10–30%) and national insurance coverage (70−90%) in Japan. To compare cost-effectiveness, we developed a novel risk score-based protocol using multivariate Cox proportional hazards regression. Patient risk scores were calculated by summing the number of independent risks suggested in the multivariate analysis, and the patients were stratified as low (0–2), intermediate (3–4), and high (5–12) risk. Next, we developed our ad-hoc surveillance protocol that enhances cost-effectiveness without increasing the number of patients who are not detected by oncological screening based on the frequency of recurrences. The estimated cost per detected recurrence in the pathology-based and risk score-based protocols was compared.

### Statistical analysis

Statistical analysis was performed using SPSS version 24.0 (IBM Corp. Released 2016. IBM SPSS Statistics for Windows, Version 24.0. Armonk, NY, USA: IBM Corp.) and GraphPad Prism 5.03 (GraphPad Software, San Diego, CA, USA). Categorical variables were compared using Fisher exact test or the chi-square test. Differences between groups were compared using *t* tests or Mann–Whitney *U* test. The Kruskal–Wallis test was used to compare medians among the 3 groups. All tests were 2-sided, and a *P* value < 0.05 was considered statistically significant. Recurrence-free survival in patients with recurrence stratified by risk criteria was estimated using the Kaplan–Meier method and compared by the log-rank test. Uni- and multivariate Cox proportional hazards regression were used to identify factors independently associated with recurrence-free survival; hazard ratios (HRs) with 95% confidence intervals (CIs) were calculated after simultaneously controlling for potential confounders including patient demographic and clinicopathological variables.

### Ethical standards

This study was performed in accordance with the ethical standards of the Declaration of Helsinki and approved by an ethics review board of Hirosaki University School of Medicine (authorization numbers 2017–089).

### Informed consent

For this type of study, formal written consent is not required. Pursuant to the provisions of the ethics committee and the ethic guideline in Japan, written consent was not required in exchange for public disclosure of study information in the case of retrospective and/or observational study using a material such as the existing documentation. The study information was open for the public consumption at http://www.med.hirosaki-u.ac.jp/∼uro/html/IRB/IRBdoc.html.

## SUPPLEMENTARY MATERIALS FIGURE



## Abbreviations

UTUC: upper tract urothelial carcinoma
RNU: radical nephroureterectomy
LVI: lymphovascular invasion
SM: surgical margin
ECOG PS: Eastern Cooperative Oncology Group Performance Status
HTN: hypertension
CVD: cardiovascular disease
DM: diabetes mellitus
eGFR: estimated glomerular filtration rate
CKD: chronic kidney disease
NAC: neoadjuvant chemotherapy
HR: hazard ratio
95% CI: 95% confidence interval
