# Supplementary material for: Risk-stratified surveillance protocol improves cost-effectiveness after radical nephroureterectomy in patients with upper tract urothelial carcinoma
Source: Oncotarget. 2018 May 1;9(33):23047–57. doi: 10.18632/oncotarget.25198 (PMC5955429; doi:10.18632/oncotarget.25198)
Supplement: Supplementary file 1 [file oncotarget-09-23047-s001.pdf]

Risk-stratified surveillance protocol improves cost-effectiveness after radical nephroureterectomy in patients with upper tract urothelial carcinoma

SUPPLEMENTARY MATERIALS

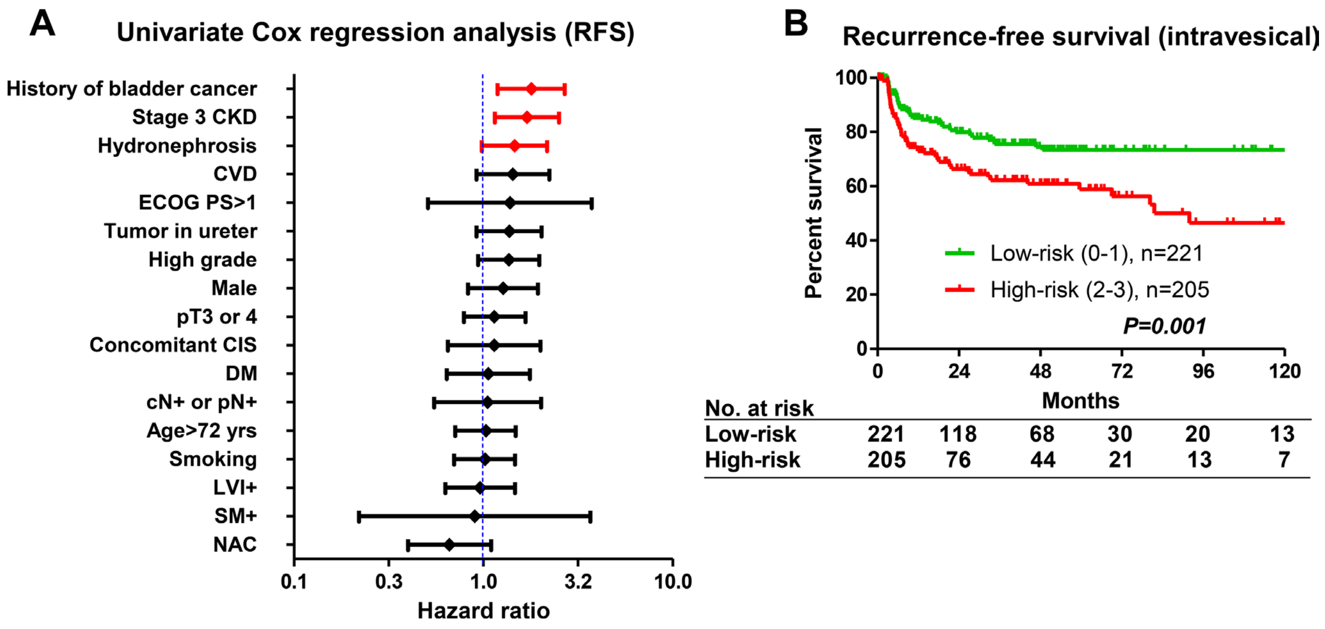

Supplementary Figure 1: Univariate Cox regression analysis and risk stratification for intravesical recurrence. (**A**) Univariate Cox regression analysis for intravesical recurrence-free survival in patients with radical nephroureterectomy (RNU). Positive history of bladder cancer and preoperative stage 3 chronic kidney disease (CKD) were independent factors for intravesical recurrence. Although presence of hydronephrosis was not significant ($P = 0.062$), we included it as a risk score calculation because of marginal P value. (**B**) Recurrence-free survival in patients with risk scores 2–3 (high-risk) was significantly shorter than in those with risk scores 0–1 (low-risk) ($P = 0.001$).
